# Visual pigment chromophore usage in Nicaraguan Midas cichlids: phenotypic plasticity and genetic assimilation of *cyp27c1* expression

**DOI:** 10.1007/s10750-024-05660-w

**Published:** 2024-08-02

**Authors:** César Bertinetti, Axel Meyer, Julián Torres-Dowdall

**Affiliations:** 1 Zoology and Evolutionary Biology, Department of Biology, University of Konstanz, Constance, Germany; 2 Department of Biological Sciences, University of Notre Dame, Notre Dame, IN USA

**Keywords:** *cyp27c1* gene expression, Sensory ecology, Visual plasticity, Neotropical cichlids

## Abstract

The wide-ranging photic conditions found across aquatic habitats may act as selective pressures that potentially drive the rapid evolution and diversity of the visual system in teleost fish. Teleost fish fine-tune their visual sensitivities by regulating the two components of visual pigments, the opsin protein and the chromophore. Compared with opsin protein variation, chromophore usage across photic habitats has received little attention. The Nicaraguan Midas cichlid species complex, *Amphilophus* cf *citrinellus* [Günther 1864], has independently colonized seven isolated crater lakes with different photic conditions, resulting in several recent adaptive radiations. Here, we investigate variation in *cyp27c1*, the main enzyme modulating chromophore exchange. We measured *cyp27c1* expression in photic environments in the wild, its genetic component in laboratory-reared fish, and its response to different light conditions during development. We found that photic environments significantly predict variation in *cyp27c1* expression in wild populations and that this variation seems to be genetically assimilated in two populations. Furthermore, light-induced *cyp27c1* expression exhibited genotype-by-environment interactions in our manipulative experiments. Overall, within-lake variation in *cyp27c1* expression was higher and inversely related to variation in opsin gene expression along the photic gradient, emphasizing the key role of *cyp27c1* in the visual ecology of cichlid fish.

**Supplementary Information:**

The online version contains supplementary material available at 10.1007/s10750-024-05660-w.

## Introduction

Cichlid fishes (Cichlidae) are the most species-rich family of vertebrates, inhabiting a wide range of aquatic habitats in tropical regions (Stiassny & Meyer, 1999; Barlow, 2000; Turner, 2007; Henning & Meyer, 2014). Cichlid biodiversity comprises remarkable examples of adaptive radiations diverging in phenotypic traits such as pharyngeal jaws, body coloration or the visual system (Meyer, 1993; Schluter, 2000; Fan et al., 2012; Brawand et al., 2014; Franchini et al., 2014; Kusche et al., 2015; Singh et al., 2022). Given that cichlids occupy a large range of aquatic light environments (e.g., turbid rivers, clear springs, blackwater streams, clear crater lakes, eutrophic lakes), studying their visual ecology allows us to understand the environmental and evolutionary drivers of biological diversity (Lythgoe, 1979; Carleton et al., 2016; Hauser et al., 2021; Torres‐Dowdall et al., 2021). Visual traits in cichlid fish have diversified in response to selective pressures imposed by ambient light conditions (Spady et al., 2005; Sugawara et al., 2005; O'Quin et al., 2010; Nandamuri et al., 2017; Torres-Dowdall et al., 2017; Härer et al., 2018; Escobar-Camacho et al., 2019; Musilova et al., 2019; Hauser et al., 2021), feeding ecology (Hofmann et al., 2009; Irisarri et al., 2018; Ricci et al., 2023), lineage-specific factors (Torres-Dowdall et al., 2015) or mating preferences (Seehausen et al., 2008; Schneider et al., 2020). Both phenotypic plasticity and genetic adaptation are mechanisms known to drive variation in cichlids and their visual traits (Meyer, 1987, 1989; Carleton et al., 2016; Nandamuri et al., 2017; Carleton & Yourick, 2020). The small adaptive radiation of the Midas cichlid species complex *Amphilophus* cf. *citrinellus* [Günther 1864] from the Nicaraguan great and crater lakes represents an ideal system for studying the contributions of both environmental and genetic factors to the evolution of visual traits (Torres-Dowdall et al., 2017; Härer et al., 2018; Karagic et al., 2018, 2022; Torres-Dowdall & Meyer, 2021; Bertinetti et al., 2024a). Midas cichlids from the great lakes Managua and Nicaragua have independently colonized multiple crater lakes within the last 800–4700 years (Barluenga et al., 2006; Elmer et al., 2014; Kautt et al., 2016, 2018, 2020). While great lakes are characterized by high turbidity, crater lakes encompass rather clear (e.g., Apoyo, Xiloá, Apoyeque, As. Managua) and increasingly turbid habitats (e.g., Masaya, As. León, Tiscapa) distributed along a photic gradient (Elmer et al., 2010; Torres-Dowdall & Meyer, 2021; Bertinetti et al., 2024a). Therefore, within each lake along this photic gradient, an extensive range of gradually varying photic environments is represented, ranging from clear, spectrally broad, luminous habitats to turbid, spectrally narrow, and dim conditions. The selective pressures imposed by these novel photic environments have driven the evolution of color vision in Midas cichlids (Torres-Dowdall et al., 2017).

Vision in cichlids is initiated when photons reaching cone photoreceptor cells in the retina trigger the isomerization of chromophores within the visual pigments (Palczewski et al., 2000; Shichida & Matsuyama, 2009; Cronin et al., 2014). The likelihood of a photon of a certain wavelength being absorbed by the visual pigment depends on both the amino acid sequence of its opsin protein and the type of chromophore (Lythgoe, 1979; Govardovskii et al., 2000; Cronin et al., 2014). The spectral absorbance of the visual pigments ultimately determines the spectral sensitivity of the photoreceptor and provides the basis for color vision (Munz & McFarland, 1977; Lythgoe, 1979). While the opsin repertoire determines the spectral class of visual pigments, chromophore exchange allows spectral tuning by shifting sensitivity towards short or long wavelengths (Dartnall & Lythgoe, 1965; Hawryshyn & Hárosi, 1994). Chromophore switching is a key mechanism for modulating spectral sensitivities and is thus expected to evolve in response to selective pressures imposed by ambient light conditions (Crescitelli et al., 1985; Bowmaker, 1990; Loew, 1995; Partridge & Cummings, 1999; Douglas & Djamgoz, 2012). Fish typically possess two chromophore types; vitamin A_1_ derived, 11-*cis* retinal, referred here as A_1_, or vitamin A_2_ derived, 11-*cis* 3,4*-*didehydroretinal, referred here as A_2_ (Wald, 1961; Hárosi, 1994; Cronin et al., 2014). Based on their spectral properties, A_2_ chromophores tend to be predominant in turbid freshwater habitats, potentially enhancing the fine-tuning of the visual system by broadening and shifting the peak sensitivity of visual pigments towards longer wavelengths (Bridges, 1972; Corbo, 2021). For instance, diadromous species, such as salmon or eels, are known to rely on A_1_ chromophores during their marine stage and switch to A_2_ as they migrate to freshwater habitats (Wald 1941; Beatty, 1975).

Although most freshwater fish use A_2_ chromophores, factors such as seasonality, developmental stage, and habitat affect the ratio of A_1_/A_2_ in the retina (Wald, 1937; Crescitelli, 1958; Temple et al., 2006). These findings were based on microspectrophotometry (MSP), which is considered the gold standard for measuring the absorbance of visual pigments bound to distinct chromophores (Liebman, 1972). MSP studies have revealed that many shallow marine fish species use A_2_ chromophores, and that A_1_ chromophores are also often found in freshwater fish, e.g., cichlid fish from Lake Malawi (Muntz, 1976; McFarland, 1977; Cummings & Partridge, 2001; Munz & Toyama et al., 2008). The patterns of chromophore usage across different taxa suggest that spectral tuning via chromophore switching is not a discrete mechanism (e.g., marine vs. freshwater, turbid vs. clear) but might indeed allow for gradual phenotypic variation that arises in response to small-scale environmental changes. Therefore, understanding the environmental drivers of chromophore ratios in fish requires both the study of populations experiencing a gradient of conditions and an easily quantifiable estimate of chromophore usage. Midas cichlids have diverged in their visual system along a range of gradually varying photic environments. Fish in more turbid environments were shown to express more long wavelength opsin genes than those in clear lakes. The magnitude and directionality of visual tuning in Midas cichlids via opsin gene expression are significantly predicted by local photic conditions (Bertinetti et al., 2024a). Importantly, there is also significant variation in A_1_/A_2_ usage (Torres-Dowdall et al., 2017). However, the magnitude of the variation in chromophore usage along the photic gradient and its genetic component remain unknown.

The molecular mechanisms underlying the conversion of vitamin A_1_ to A_2_ have been well established over the last decade (Enright et al., 2015; Morshedian et al., 2017; Corbo, 2021). Although the existence of different chromophores has been known since the past century (Wald, 1937), candidate genes involved in chromophore exchange remained elusive until transcriptomic data from the retinal pigment epithelium (RPE) of zebrafish *Danio rerio* [Hamilton 1822] pointed to a key P450 enzyme, namely *cyp27c1* (Enright et al., 2015). The role of *cyp27c1* in synthesizing vitamin A_2_ was further experimentally proven by knockout mutants in zebrafish and electrophysiological measurements in the sea lamprey *Petromyzom marinus* [Linnaeus 1758] (Morshedian et al., 2017). Hence, the expression of *cyp27c1* is tightly linked to the abundance of A_2_ chromophores and allows organisms to fine-tune their chromophore ratios via gene expression (Corbo, 2021). In Midas cichlids, expression of *cyp27c1* is increased in both turbid great lakes populations Nicaragua and Managua compared to the clearest crater lakes Apoyo and Xiloá (Torres-Dowdall et al., 2017). Moreover, increased *cyp27c1* expression was shown to correlate with A_2_ chromophore usage measured by MSP, emphasizing the role of *cyp27c1* as a molecular mechanism behind chromophore switching. Additionally, this pattern of variation in *cyp27c1* expression in fish from the turbid great lakes vs clear water crater lakes was also observed in six species of cichlids with distinct ecologies inhabiting Lakes Managua and Xiloá, suggesting strong convergence in chromophore usage due to common environmental pressures (Härer et al., 2018). However, how the gradient of photic conditions inhabited by Midas cichlids influences variation in *cyp27c1* expression in distinct populations remains to be determined.

To understand the intrinsic and extrinsic factors influencing chromophore usage in Midas cichlids, we measured the expression of *cyp27c1* in both wild-caught and laboratory-reared adult fish from seven crater lakes, two great lakes and one riverine population of Nicaraguan Midas cichlids. Additionally, to understand the effect of light conditions on chromophore usage, we raised offspring from two crater lakes and one older great lake source population of Midas cichlids under two distinct light treatments and measured their *cyp27c1* expression. Specifically, our study aims (i) to determine the variation of *cyp27c1* expression in the wild in response to the photic gradient of the Nicaraguan lakes, (ii) to study the genetic component of *cyp27c1* variation by determining the pattern of gene expression under standardized rearing conditions (i.e., common garden), and (iii) to test the role of light environments on *cyp27c1* expression by experimentally manipulating light treatments in the laboratory. By combining measurements of photic conditions at ten locations inhabited by Midas cichlids with the expression of *cyp27c1* in adult fish from these ten locations, we show a fine-scale pattern of variation in chromophore usage that has both genetic and plastic components.

## Materials and methods

### Experimental design

To measure the variation in *cyp27c1* expression in wild populations and estimate the genetic component of this variation, wild-caught (wild) and F3 laboratory-reared (lab) individuals of the Midas cichlid species complex *Amphilophus* cf. *citrinellus* were used in this study. Wild-caught adults were collected using gill nets from ten locations within the Nicaraguan great and crater lakes as described by Bertinetti et al. (2024a). All laboratory-reared fish were raised under standardized lighting conditions with a 12L:12D photoperiod following housing protocols from the animal facility of the University of Konstanz (i.e., common garden conditions). Our study included adult fish from two great lakes: Managua (wild = 8, lab = 8) and Nicaragua (wild = 8, lab = 8); seven crater lakes: Apoyo (wild = 7, lab = 5), Apoyeque (wild = 8, lab = 6), Asososca Managua (wild = 8, lab = 6), Asososca León (wild = 8, lab = 6), Masaya (wild = 8, lab = 9), Tiscapa (wild = 8, lab = 6) and Xiloá (wild = 8, lab = 7); and one riverine population River San Juan (wild = 8, lab = 0). To further understand the role of light conditions in the variability of *cyp27c1* expression, we raised F3 descendants of wild-caught Midas cichlid from two crater lakes, Apoyo and Xiloá, and the great lake Managua under two different light treatments. One light treatment simulated the broad spectrum found in clear crater lakes (i.e., white light), and the other simulated the long wavelength-shifted spectrum of the turbid great lakes (i.e., red light). Eggs were collected from the animal facility at the University of Konstanz between November 2020–July 2021. Upon hatching, ~ 80 individuals per species were divided into two groups and randomly assigned to one of the two light treatments, where they were maintained until day 220.

### Photic environments in the wild and laboratory light treatments

To investigate the role of ambient light conditions on *cyp27c1* gene expression, we used measurements of underwater spectral irradiance from Bertinetti et al. (2023) for all locations where fish were collected (Fig. S1). To characterize the photic conditions experienced by Midas cichlids at their lake of origin, we used principal component analysis (PCA) to generate the main composite axis of the photic environments, as described by Bertinetti et al. (2024a). Briefly, normalized irradiance at 1 m depth, i.e., the number of photons per second per square centimeter at a given wavelength divided by its maximum, was used to estimate photic variables. The photic variables consisted of the spectrum halving wavelength, *λP*_50_, and the wavelengths at which 25% and 75% of the photons are located, *λP*_25_ and *λP*_75_, respectively (McFarland & Munz, 1975; Mobley, 1994). Measurements of both sidewelling and downwelling (i.e., horizontal and vertical orientations of the sensor, respectively) were used to estimate the photic variables. We also included the percentage of downwelling irradiance at 1 m depth compared with the water surface, %*E*_*d*_. Summary statistics and biplot showing the loadings of each photic variable on PCA are reported in the supplementary material (Table S1, Fig. S2).

Using the spectral properties of the photic environments in the Nicaraguan lakes as a reference, we simulated a clear crater lake-like environment (i.e., broadband, higher proportion of short wavelength—white light) and a turbid great lake-like environment (i.e., spectrally narrow, long-wavelength enriched—red light) to raise newly hatched offspring of Midas cichlid fish (Fig. S3A). For this, fish were raised at 25 °C in 7 l aquarium tanks placed within light cabinets containing either white light (Cree XP-G3 S5 SMD-LED, Lumitronix, Germany) or red light (Nichia NCSRE17AT SMD-LED, Lumitronix, Germany) with 12L:12D photoperiod (Fig. S3B). Fish were fed *live brine shrimp (Artemia sp.) and water fleas (Daphnia magna) *ad libitum*.* At the age of 220 dph, fish were euthanized using an overdose of MS-222, and their eyes were dissected and stored in RNAlater (Sigma Aldrich, MO, USA) at − 20 °C until RNA extraction. All fish were collected between 1 and 3 pm to control for circadian patterns in gene expression (Halstenberg et al., 2005; Yourick et al., 2019).

### *cyp27c1* gene expression

Two-step reverse transcription quantitative real-time PCR (RT-qPCR) was used to measure the expression of *cyp27c1* in Midas cichlids. A standard acid-guanidinium-phenol–chloroform protocol was used to extract RNA from eye tissues. For this, eyes were homogenized in 1 ml TRI® Reagent (Molecular Research Center, OH, USA) placed in lysing matrix D (MP Biomedicals, CA, USA) using a tissue homogenizer for 30 s, 3500 rpm at 25 °C (Powerlyzer 24, Qiagen, Hilden, Germany). After this, 200 µL acidic chloroform was mixed, incubated at room temperature for 10 min, and centrifuged for 15 min at 13,000 rpm, 4 °C. Then, 400 µL of the aqueous phase was precipitated in the same volume of isopropanol, incubated for 10 min on ice and centrifuged for 8 min at 13,000 rpm, 4 °C. After discarding the supernatant, 1 ml of 75% ethanol was added and centrifuged for 5 min at 13,000 rpm, 4 °C. The RNA pellet was then air-dried for 3 min at room temperature and resuspended in 40 µL of nuclease-free water. Qubit 4 Fluorometer (Fischer Scientific, NH, USA) and 4200 Tapestation (Agilent, CA, USA) were used to quantify and assess the quality of the RNA, respectively. A total of 200 ng of RNA was used to synthesize first-strand cDNA using GoScript™ Reverse Transcription System (Promega, WI, USA). Expression of *cyp27c1* and two reference genes, *gapdh2* and *imp2,* was measured for 40 cycles at 95 °C for 15 s, 60 °C for 1 min, and an initial denaturation step at 95 °C for 2 min (CFX Duet, Bio-Rad Laboratories, CA, USA). Each reaction was assembled following the manufacturer’s protocol with 2 µl template cDNA, 0.5 µl forward primer (10 µM), 0.5 µl reverse primer (10 µM), 10 µl GoTaq RT-qPCR Master Mix 2x (Promega, WI, USA), and 7 µl nuclease-free water. Primer sequences and amplification efficiencies are reported in the supplementary material (Table S2). The mean quantification cycle (*Cq*) from three technical replicates was used for further analysis. The expression of *cyp27c1* was normalized using the geometric mean of two reference genes, *gapdh2* and *imp2* (REF), as follows

1 $$R{Q_{cyp27c1}} = {E_{cyp27c1}}^{\left( {C{t_{REF}} - ~C{t_{cyp27c1}}} \right)}$$

### Statistical analysis

To predict the variation in *cyp27c1* across populations based on the native photic environment, we used a linear mixed-effect model with log2-transformed relative expression of *cyp27c1* as the response variable*,* photic axis (PC1) as the predictor variable, and lake as a random intercept. The diagnostic plots are provided in the Supplementary Material (Fig. S4). Subsequently, we performed ANOVA (type II) to test for the overall effects of PC1 on *cyp27c1* expression. Furthermore, we used two-way ANOVA to estimate the effect of species identity and rearing light environment on *cyp27c1* expression in juvenile Midas cichlids. Given the results seen from wild-caught and laboratory-reared fish, our interest was in the interaction between photic conditions and the population of origin (i.e., G × E interactions). We complemented this general test with a Tukey’s HSD post-hoc comparison test, focusing on the effect of light conditions within the species. Diagnostic plots are provided in the supplemental materials (Fig. S4). Additionally, to estimate the influence of photic conditions on intra-population variability in *cyp27c1* expression, we regressed the coefficients of variation of our estimates of log2-transformed relative *cyp27c1* expression against the photic axis (PC1). All statistical analyses were performed in R (R Core Team, 2020).

## Results

### In the wild, expression of *cyp27c1* increases in environments shifted towards longer wavelengths

**Fig. 1:**
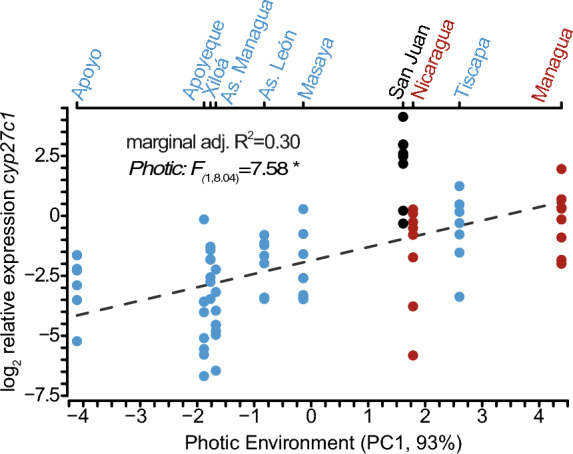
Log2-transformed relative expression of *cyp27c1* in wild-caught Midas cichlid species was predicted based on the photic environment at the lake of origin. The dashed line shows the mean regression line of the mixed-effects model, with the lake of origin as a random intercept and the photic environment (PC1) as the predictor variable. The upper-left corner shows the *F*-values (ANOVA Type II) and marginal adj. *R*^2^, the adjusted proportion of variance explained only by PC1 (30%). Great lakes are shown in red, crater lakes in blue and River San Juan in black. Light measurements obtained from Bertinetti et al. (2023). *p* < 0.05 = ‘*’

To estimate variation in chromophore usage among Midas cichlid species in the wild, we measured *cyp27c1* gene expression in wild-caught individuals across the photic gradient of the Nicaraguan lakes. Lakes within this gradient range continuously from clear, broad-spectrum, bright conditions (e.g., clear crater lakes Apoyo and Xiloá) to dark, spectrally narrow, dim habitats (e.g., the turbid great lakes or eutrophic crater lake Tiscapa; Fig. S1). Approximately 93.5% of the variance among photic environments (PC1) was explained by photon distribution, *λP-*values, that is, spectra being rather short- or long-wavelength shifted (Table S1, Fig. S2). Using a linear mixed effect model with the lake of origin as a random effect, we found a significant effect of the photic environment on log2-transformed *cyp27c1* expression, *F*_1,8.04_ = 7.58, *p* = 0.024, which explained 30% of the phenotypic variation in our model (Fig. 1). Broadly, as the environment becomes dimmer, long-wavelength shifted, and spectrally narrow, the expression of *cyp27*c1 increases. Hence, more vitamin A_1_ is expected to be transformed into vitamin A_2,_ consequently increasing the usage of A_2_ chromophores in habitats shifted towards longer wavelengths. Thus, the variation observed in the wild matches the predictions based on expected chromophore usage, with increasing expression levels of *cyp27c1* in Midas cichlids experiencing turbid conditions.

### Within lake variation in *cyp27c1* is explained by photic conditions

**Fig. 2:**
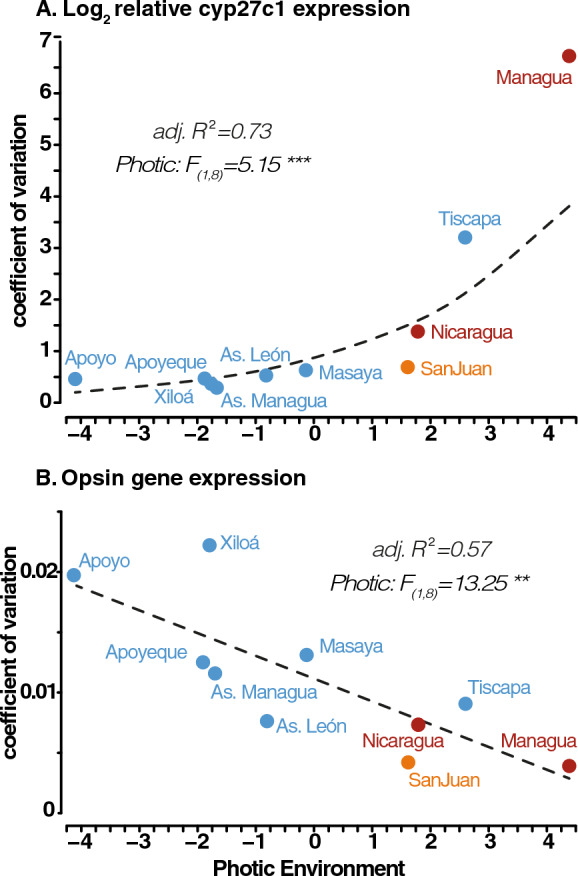
**A** Exponential regression of coefficients of variation of log2-transformed *cyp27c1* relative expression within each population for wild-caught fish in response to ambient photic environment (PC1) **B** Linear regression of coefficients of variation in predicted sensitivity index (PSI) in response to ambient photic environment (PC1) based on data from Bertinetti et al. (2024a). *F*-value (ANOVA II) and adjusted R-squared reported in upper corners. *p* < 0.001 = ‘***’, *p* < 0.01 = ‘**’

To estimate the degree of variability in chromophore usage within populations, we regressed the coefficients of variation in *cyp27c1* expression against the photic axis (PC1, Table S1, Fig. S2). Variation in *cyp27c1* expression was significantly predicted by photic conditions, *F*_1,8.04_ = 5.15, *p* < 0.001, and was better explained by an exponential fit (adj. *R*^2^ = 0.73), with coefficients of variation increasing sharply as habitats become long-wavelength shifted (Fig. 2A). Furthermore, coefficients of variation (mean ± SE) for log2 relative *cyp27c1* expression averaged 1.47 ± 0.64, which were significantly higher than those for predicted sensitivity index values based on opsin gene expression(0.01 ± 0.002; Fig. 2B). This suggests that expression in *cyp27c1* is more variable than opsin gene expression (Mann Whitney *U* Test, *W* = 100 *p* < 0.001).

### Inter-population variation in *cyp27c1* expression is reduced under common garden conditions

**Fig. 3:**
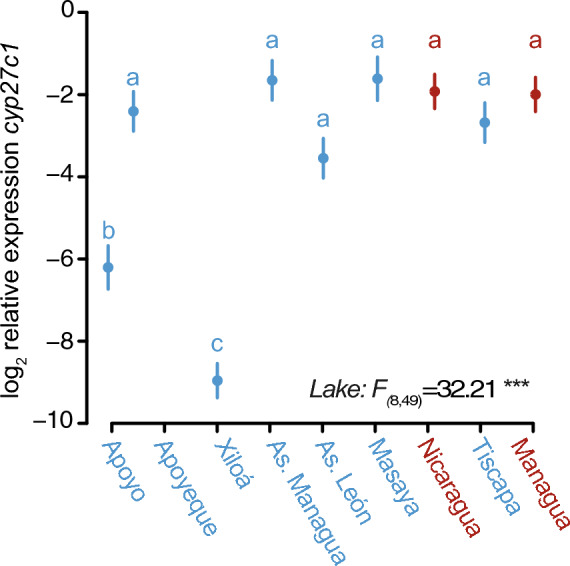
Log2-transformed relative expression of *cyp27c1* differs significantly across populations in laboratory-reared Midas cichlid. The lower right corner shows the *F*-values (ANOVA Type II). Letters indicate groups based on Tukey’s HSD test for multiple comparisons. Dots and arrows indicate mean values and standard errors, respectively. Great lakes are shown in red and crater lakes in blue. *p* < 0.001 = ‘***’

To understand the genetic component of the variation in *cyp27c1* expression across populations of Midas cichlids, we measured the expression of *cyp27c1* in F3 individuals reared under common garden conditions. Log2-transformed relative expression of *cyp27c1* was significantly different across populations based on one-way ANOVA, *F*_1,8_ = 7.58, *p* < 0.001 (Fig. 3). Post-hoc comparison using Tukey’s HSD showed that mean values in log2-relative *cyp27c1* in crater lake populations from Xiloá and Apoyo were significantly lower than all other populations. Hence, variation in *cyp27c1* expression is not maintained under common garden conditions, except for Xiloá and Apoyo, where populations have diverged significantly from their ancestral great lake populations Managua and Nicaragua, respectively.

### Genotype-by-environment interactions contribute to inter-population differences in *cyp27c1* expression

**Fig. 4:**
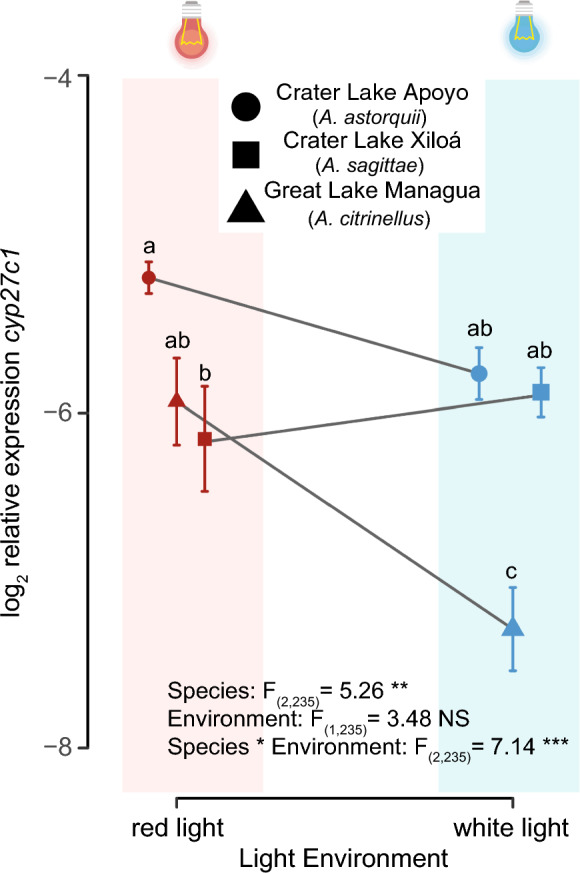
Log2-transformed relative expression of *cyp27c1* shows a significant genotype-by-environment interaction in three laboratory-reared populations of Midas cichlids (crater lakes Apoyo and Xiloá, and great lake Managua). Text in the lower part of the figure panel shows *F*-values (ANOVA Type III). Letters display groups based on Tukey’s HSD test for multiple comparisons. Dots and arrows indicate mean values and their standard errors, respectively. Fish reared under red light are shown in red, whereas fish reared under white light are shown in blue. NS = not significant, *p* < 0.01 = ‘**’*p* < 0.001 = ‘***’

To understand the effect of light conditions on variation in *cyp27c1* expression observed in Midas cichlids, we reared newly hatched F3 individuals from great lake population, Managua, and two crater lake populations, Xiloá and Apoyo under two different light conditions and measured expression of *cyp27c1* in juveniles (age of 220 dph). Two-way ANOVA was used to determine the effects of species identity and rearing light environment on *cyp27c1* gene expression*.* We found a significant interaction between species and light environment, *F*_2,235_ = 7.14, *p* < 0.001 (Fig. 4). This confirms the variation among species in their response to photic conditions (i.e., G × E interaction). Further, post-hoc comparison using Tukey’s HSD test showed that log2-relative *cyp27c1* expression was only significantly different between light treatments in fish from Great Lake Managua. Neither of the two crater lake species showed significant differences between fish reared under white or red light. Hence, while the ancestral great lake populations responded plastically to the light treatment, neither of the two crater lake populations displayed a significant environmental effect on their reaction norms.

## Discussion

In this study, we examine variation in *cyp27c1* expression among Nicaraguan Midas cichlids inhabiting a broad range of photic conditions to understand the genetic and environmental components of chromophore usage in fish. We measure gene expression of *cyp27c1*, a molecular switch for chromophore exchange, both in the wild and under common garden conditions across populations experiencing different native photic habitats. Expression of *cyp27c1* in the wild results in gradual phenotypic variation, suggesting that chromophore usage may be fine-tuned to local environmental conditions (Fig. 1). Additionally, intra-population variation in *cyp27c1* expression increased exponentially as environments become more spectrally narrow and long-wavelength-shifted, with *cyp27c1* expression being more variable among individuals and showing an opposite pattern than opsin gene expression (Fig. 2). However, most variation in *cyp27c1* expression was lost under common garden conditions, except for two crater lake populations, Apoyo and Xiloá (Fig. 3). Fish from clear crater lakes Apoyo and Xiloá show genetic divergence from the rest with lower expression of *cyp27c1* in the absence of habitat-specific photic cues. The manipulative experiments showed a clear genotype-by-environment interaction in *cyp27c1* expression. While fish from Apoyo and Xiloá showed no significant plasticity in response to different light treatments, individuals from Great Lake Managua showed increased *cyp27c1* expression in red-shifted environments (Fig. 4). Collectively, our findings highlight the role of chromophore usage as a relevant source of variation in visual sensitivity across photic conditions, enabling Midas cichlids to track environmental conditions via genetic evolution and plastic responses.

Diversity in the visual system of cichlid fishes in the wild has been studied across many species of both African and Neotropical lineages (reviewed in Carleton et al., 2016; Carleton & Yourick, 2020). Multiple studies have found significant associations between opsin gene expression or opsin sequence divergence and photic habitats under contrasting conditions, for example, shallow vs. deep (Sugawara et al., 2005; Hahn et al., 2017; Ricci et al., 2022) or turbid vs. clear (Torres-Dowdall et al., 2017; Härer et al., 2018; Escobar-Camacho et al., 2019). A small number of studies have also examined the variation in opsin genes under gradually varying photic conditions (Seehausen et al., 2008; Bertinetti et al., 2024a). However, chromophore usage, one of the two components of visual pigments, together with opsins, has received relatively little attention when investigating the visual ecology of fish (Corbo, 2021). This apparent lack of studies might be because quantitative measurements of chromophore usage require techniques such as MSP or High-Performance Liquid Chromatography (HPLC) which are costly and difficult to implement in the field. Instead, we used RT-qPCR to measure the expression of *cyp27c1,* which is necessary and sufficient to catalyze the conversion of 11-cis retinal (A_1_) or 11-*cis* 3,4-didehydroretinal (A_2_). We find substantial variation in *cyp27c1* expression in Midas cichlids both between and within populations, which is expected to be translated into variation in chromophore usage (Corbo, 2021; Enright et al., 2015). However, the extent to which levels of *cyp27c1* expression reflect levels of A_2_ usage in visual pigments remains understudied and requires correlating gene expression data with MSP data from different species of the Midas cichlid complex (for example, Torres-Dowdall et al. 2017).

Our analysis indicates that approximately one-third of the variation in *cyp27c1* expression can be attributed to differences in photic conditions. Although this is a significant percentage of the variation in *cyp27c1*, other factors not considered here might affect expression levels of *cyp27c1* in the wild. For instance, fish from the riverine population San Juan showed higher levels of *cyp27c1* expression than expected based on their photic conditions (Fig. 1). High relative expression of *cyp27c1* expression was also previously reported for Midas cichlids from River San Juan, but the pattern was not consistent for the other six species of cichlids analyzed in that study (Härer et al., 2018). This suggests that habitat-specific and species-specific factors might influence the large amount of unexplained variance that was not due to photic conditions in our model. Alternatively, the temporal dynamics of *cyp27c1* are not captured by our study, since our data represents a snapshot of the fluctuating environments encountered in nature through time (Bridges, 1964; Temple et al., 2006; Corbo, 2021; Foster et al., 2024). Therefore, future studies should consider the seasonality and diurnal patterns of chromophore usage and how fast chromophore exchange in the retina is mediated by the expression levels of *cyp27c1* across populations. Nonetheless, the significant association between *cyp27c1* expression and ambient light conditions shows that *cyp27c1* tracks local photic conditions. Hence, caution is advised when interpreting patterns of expected variation in visual sensitivity in studies where *cyp27c1* expression is not quantified, thereby neglecting the potential role of chromophore usage variation. Therefore, we suggest that future studies addressing the visual ecology of teleost fishes should not only focus on opsin genes, but also include measurements of *cyp27c1*. Studying how changes in the environment relate to patterns in *cyp27c1* expression and its temporal dynamics remains key to understanding the visual ecology of chromophore exchange in aquatic environments.

In addition to the role of photic environments in shifting the mean expression of *cyp27c1* in Midas cichlid populations, we find that also within-lake variation in *cyp27c1* is influenced by photic conditions (Fig. 2A). Populations inhabiting clear crater lakes show little variation in *cyp27c1* expression which increases exponentially as habitats become more turbid. Interestingly, the pattern of *cyp27c1* variability contrasts in magnitude and directionality with that reported for opsin gene expression in the same fish (Fig. 2B, Bertinetti et al. 2024a). Overall, the coefficients of variation in *cyp27c1* expression were higher than those for opsin gene expression and in the opposite direction, counteracting the linear increase in intra-population opsin gene expression variation as the environment became clearer. Hence, our results support the idea that chromophore usage mediated via cyp27c1 expression is more variable and prone to rapid fine-tuning of visual sensitivities than opsin gene expression. More importantly, the contrasting patterns of variation in *cyp27c1* and opsin gene expression suggest that photic conditions influence the predominant mechanism used to modulate visual sensitivities. In clear, short-wavelength-shifted, and spectrally broad environments, populations of Midas cichlids show high variation in opsin gene expression but little variation in *cyp27c1* expression. This implies that in clear habitats, opsin gene expression is highly variable, but most visual pigments are bound to A_1_ chromophores. In contrast, in dark, long-wavelength-shifted, spectrally narrow environments, individuals exhibit little variation in opsin gene expression, but differ widely in their *cyp27c1* expression. Therefore, under turbid conditions, chromophore usage might be the predominant mechanism behind the fine-tuning of visual sensitivities, with a stable ratio of opsin genes bound to highly flexible ratios of A_1_/A_2_. If the use of a “variable opsin—stable chromophore” versus “stable opsin—variable chromophore” strategy to modulate visual sensitivities is a particular trait of Midas cichlid or a generalizable biological feature of teleost visual systems, it will require the study of more species inhabiting similar photic gradients.

The patterns of variation in *cyp27c1* expression in laboratory-reared Midas cichlids suggest that the variation observed among populations in the wild lacks a strong genetic component. Under common garden conditions, the range of variation in *cyp27c1* expression was greatly reduced, with only two crater lake populations, Apoyo and Xiloá, differing from the others. These populations are among the oldest crater lake colonization events by Midas cichlids, Apoyo 4700 years ago from Great Lake Nicaragua, and Xiloá ~ 4300 years ago from Great Lake Managua (Kautt et al., 2020). Given the drastic differences in photic conditions and the longer exposure to the novel environment in fish from Apoyo versus Nicaragua and Xiloá versus Managua, previous studies have shown parallel evolution in opsin gene expression and chromophore usage in these populations (Torres-Dowdall et al., 2017). In contrast, our study suggests a predominant role for phenotypic plasticity instead of local adaptation as the main modulator of variation in *cyp27c1* expression between populations inhabiting gradually varying photic environments. This result is aligned with the expectation that chromophore exchange is a labile trait that enables rapid fine-tuning of visual sensitivities to match ongoing light conditions (Munz & McFarland, 1977; Morshedian et al., 2017; Corbo, 2021). While cone opsin genes determine spectral classes and therefore the broad chromaticity of the visual system, chromophore exchange allows selective shifting of sensitivities within the spectral class range towards longer or shorter wavelengths in a more fine-tuned manner (Dartnall & Lythgoe, 1965; Hawryshyn & Hárosi, 1994). Therefore, studies focusing on the phenotypic plasticity of chromophore usage and its implications for visual performance would be useful for understanding the adaptive value of *cyp27c1* variation in natural populations experiencing environmental heterogeneity. In particular, what is the plastic capacity of *cyp27c1* expression and how fast it translates to chromophore exchange in the retina in response to ambient cues remains unanswered (i.e., rate of plasticity, Burton et al., 2022; Dupont et al., 2024).

Given the relevant role of photic conditions on *cyp27c1* expression, the high degree of plasticity and the genetic component observed in Xiloa and Apoyo, it can be inferred that genetic variation for plasticity in *cyp27c1* expression is present in populations of Midas cichlids. Our study indicates that the responsiveness to photic conditions differs across populations of Midas cichlids. While fish from Great Lake Managua reacted plastically to the light treatments, fish from Crater Lakes Apoyo and Xiloá did not show a significant response, suggesting a reduced sensitivity to light conditions. This suggests genetic assimilation of light-induced *cyp27c1* expression (Waddington, 1942; Pigliucci et al., 2006; Lande, 2009). The colonization of novel clear Crater Lakes Apoyo and Xiloá by populations originating from the great lakes may have canalized the low expression of *cyp27c1*, resulting in a loss of sensitivity to photic conditions (Eshel & Matessi, 1998; Gunter et al., 2017; Pigliucci & Murren, 2003; Schneider & Meyer, 2017). The relatively low expression of *cyp27c1,* leading to preferential A_1_ chromophore usage in fish from Apoyo and Xiloá (e.g., Torres-Dowdall et al. 2017 and this study), would more constitutively shift visual sensitivities to shorter wavelengths predominant in these clear crater lakes. Other factors, such as developmental trajectories, may also contribute to differences across lakes (Bridges, 1972; Corbo, 2021; Wilwert et al., 2023). For instance, adult fish from Great Lake Managua in the wild and the lab showed similar values in *cyp27c1* expression, while this was greatly reduced in juvenile fish in our manipulative experiment. This result suggests that ontogeny may also significantly contribute to variation in *cyp27c1* expression. However, more data are required to comprehensively understand the ontogenetic trajectories of *cyp27c1* expression, more data are required. Hence, experiments investigating the progression of *cyp27c1* expression through lifespan in multiple light conditions for both crater and great lake populations are required to understand the role of ontogeny and the potential for co-option of developmental pathways to facilitate genetic assimilation.

## Conclusion

As a molecular switch directly linked to chromophore usage, *cyp27c1* is a main modulator of visual sensitivity in teleost fish (Corbo, 2021; Enright et al., 2015). The photic conditions encountered by fish in nature represent a continuum, and thus, measuring phenotypic variation in response to environmental gradients is key to our understanding of trait diversity in visual ecology. Given the photic gradient inhabited by Midas cichlids and their demographic history, studying the variation in *cyp27c1* expression among populations from the Nicaraguan great and crater lakes sheds light on the drivers of chromophore usage. We found that 30% of the variation in *cyp27c1* was predicted by the native photic environment in wild-caught individuals, and that only two out of nine populations showed a genetic component for *cy27c1* expression when reared in the laboratory. The high degree of plasticity in *cyp27c1* is also evidenced by higher levels of intra-population variation compared to opsin gene expression. The different plastic responses among populations of Midas cichlids to light conditions were also evidenced by our manipulative experiments, where only fish from the Great Lake Nicaragua differed in *cyp27c1* expression across light treatments. The significant genotype-by-environment interactions found in our study highlight that light-induced changes in *cyp27c1* potentially evolved via genetic assimilation in some populations, whereas environmental drivers account for the variation observed in other populations. Interestingly, we found that within-lake variation in *cyp27c1* increases as habitats become more turbid, a pattern that is inversely related to opsin gene expression variation, suggesting that the main mechanisms used to fine-tune visual sensitivity vary along the gradient. In clear water conditions, opsin gene expression is highly variable, with little variation in *cyp27c1* gene expression, indicating that retinas are composed of more diverse ratios of opsin genes, which are mostly bound to A_1_ chromophores. In contrast, under turbid conditions, variation in opsin gene expression is constrained, but visual pigments might vary greatly in their chromophore composition, as indicated by the higher variation in *cyp27c1* expression. Overall, this emphasizes the relevant role of variation in *cyp27c1* expression across light habitats and the need for more studies addressing how *cyp27c1* enables fine-tuning of visual sensitivity across aquatic environments.

## Supplementary Information

Below is the link to the electronic supplementary material.

Supplementary file1 (DOCX 9934 KB)

## Data Availability

The data from this study and the code associated with it have been deposited in Zenodo digital repository and are publicly available at https://zenodo.org/doi/10.5281/zenodo.10850331 (Bertinetti et al., 2024b).
