# Supplementary material for: Visual pigment chromophore usage in Nicaraguan Midas cichlids: phenotypic plasticity and genetic assimilation of cyp27c1 expression
Source: Hydrobiologia. 2024 Aug 2;852(15):3831–45. doi: 10.1007/s10750-024-05660-w (PMC12304006; doi:10.1007/s10750-024-05660-w)
Supplement: Supplementary file 1 — Supplementary file1 (DOCX 9934 KB) [file 10750_2024_5660_MOESM1_ESM.docx]

**Supplemental Information for:**

**Visual pigment chromophore usage in Nicaraguan Midas cichlids: Phenotypic plasticity and genetic assimilation of *cyp27c1* expression**

César Bertinetti^1,2^, Axel Meyer^1^, Julián Torres-Dowdall^1,2^

^1^ Zoology and Evolutionary Biology, Department of Biology, University of Konstanz, Konstanz, Germany

^2^ Department of Biological Sciences, University of Notre Dame, Notre Dame, IN, USA

Corresponding author: Julián Torres-Dowdall, Email: [torresdowdall@nd.edu](mailto:torresdowdall@nd.edu)

Table of Contents

[**Table S1.** Loadings and scores associated with principal component analysis (PCA) of photic variables across the Nicaraguan great and crater lakes. Measurements obtained from Bertinetti et al. (2024). 3](#_Toc171433756)

[**Table S2**. List of primer sequences used in this study and their amplification efficiencies for RT-qPCR. 3](#_Toc171433757)

[**Figure S1**. Reference spectral curves showing the normalized downwelling irradiance (E_d_) at one meter depth for each site. Vertical solid lines below each spectral curve represent the spectrum-halving wavelength λP50 within the colored area depicting the spectral bandwidth, where 50% of the photons are found. The intensity of the spectral bandwidths represents the relative luminosity at one meter depth (%E_d_), with darker colors meaning higher %E_d_. The shaded area displayed in the background of the crater lake inserts shows the photic environment plus λP50 (dashed line) of the respective source great lake based on Midas cichlids’ colonization history (Kautt et al. 2016; 2018; 2020). 4](#_Toc171433758)

[**Figure S2.** Biplot depicting the PCA scores generated from photic variables from all sites at one meter depth where fish were collected (Table S1). Arrows represent the loadings of each variable. 5](#_Toc171433759)

[**Figure S3.** (A) Normalized downwelling irradiance at 1m depth in crater lakes Apoyo (blue) and Xiloá (green) and in great lake Managua (red) from Bertinetti et al. 2023 (B) Normalized downwelling irradiance used in the laboratory for white light treatment (blue) and red light treatment (red). 6](#_Toc171433760)

[**Figure S4.** Diagnostics plot for linear mixed-effects model using PC1 as predictor variable of log-normalized relative *cyp27c1* expression with location as a random effect (Fig. 1) 7](#_Toc171433761)

**Table S1.** Loadings and scores associated with principal component analysis (PCA) of photic variables across the Nicaraguan great and crater lakes. Measurements obtained from Bertinetti et al., (2024).

|  | PC1 | PC2 | PC3 | PC4 | PC5 | PC6 | PC7 |
| --- | --- | --- | --- | --- | --- | --- | --- |
| Standard deviation | 2.5580 | 0.5833 | 0.2538 | 0.1848 | 0.2010 | 0.0463 | 0.0222 |
| Proportion of Variance | 0.9350 | 0.0486 | 0.0092 | 0.0057 | 0.0010 | 0.0003 | 0.0001 |
| Cumulative Proportion | 0.9302 | 0.9836 | 0.9928 | 0.9985 | 0.9996 | 0.9999 | 1.0000 |
| **Loadings:** |  | | | | | | |
| Downwelling λP50 | 0.3884 | -0.0808 | 0.2040 | 0.3984 | 0.3702 | 0.0345 | -0.7098 |
| Downwelling λP25 | 0.3849 | 0.0066 | 0.6000 | 0.3461 | -0.5005 | 0.1634 | 0.3076 |
| Downwelling λP75 | 0.3844 | -0.1363 | -0.5091 | 0.4759 | 0.2009 | 0.3003 | 0.4661 |
| Relative Luminosity | 0.3284 | -0.9272 | 0.0949 | 0.1306 | -0.0751 | 0.0218 | -0.0116 |
| Sidewelling λP50 | 0.3859 | -0.2300 | -0.1855 | 0.2668 | 0.2123 | 0.7973 | 0.1061 |
| Sidewelling λP25 | 0.3836 | -0.1961 | -0.0370 | 0.5794 | 0.3061 | 0.4578 | 0.1953 |
| Sidewelling λP75 | 0.3861 | -0.1529 | -0.3981 | 0.2660 | -0.6538 | 0.1898 | -0.3669 |
| **Positions:** |  | | | | | | |
| Crater Lake Apoyo | -4.1099 | 0.0839 | 0.0595 | 0.1029 | -0.0487 | 0.0440 | -0.0254 |
| Crater Lake Xiloa | -1.7677 | -0.4963 | -0.0529 | 0.0202 | -0.0621 | 0.0971 | 0.0295 |
| Crater Lake Apoyeque | -1.8227 | 0.0003 | -0.4139 | 0.0077 | 0.1584 | 0.0455 | 0.0268 |
| Crater Lake As. Managua | -1.6728 | 0.1219 | -0.0368 | 0.0287 | -0.1081 | 0.0119 | -0.0067 |
| Crater Lake As. León | -0.8228 | 0.2966 | 0.2937 | 0.2010 | 0.0001 | 0.0039 | 0.0046 |
| Crater Lake Masaya | -0.1394 | -0.7100 | 0.0674 | 0.3323 | 0.0737 | 0.0109 | -0.0344 |
| Crater Lake Tiscapa | 2.6019 | 1.1103 | -0.2500 | 0.1520 | -0.0645 | 0.0167 | -0.0122 |
| Great Lake Nicaragua | 1.7871 | 0.0016 | 0.2920 | 0.1993 | -0.0797 | 0.0628 | 0.0342 |
| Great Lake Managua | 4.3917 | -0.8694 | -0.2176 | 0.2599 | -0.0619 | 0.0151 | -0.0128 |
| River San Juan | 1.6133 | 0.4608 | 0.2586 | 0.1190 | 0.1928 | 0.0348 | -0.0036 |

**Table S2**. List of primer sequences used in this study and their amplification efficiencies for RT-qPCR.

| GENE | PRIMER | SEQUENCE | EFFICIENCY |
| --- | --- | --- | --- |
| *cyp27c1* | CYP27C1_F1 | GGCGGTACCATGAAGATACAAC | 95.6% |
|  | CYP27C1_R1 | ACCACGAAAAGCATCCAGAG |  |
| *imp2* | AH_IMP2_F1 | GCCTGGAGCATGTTGACC | 101.3% |
|  | AH_IMP2_R1 | CGAAGTGACGGATCTTACGG |  |
| *gapdh2* | GAPDH2_F1 | TGCCCATACAAACATCATTCC | 98.7% |
|  | GAPDH2_R1 | GGCATGTCAGATCCACCACT |  |


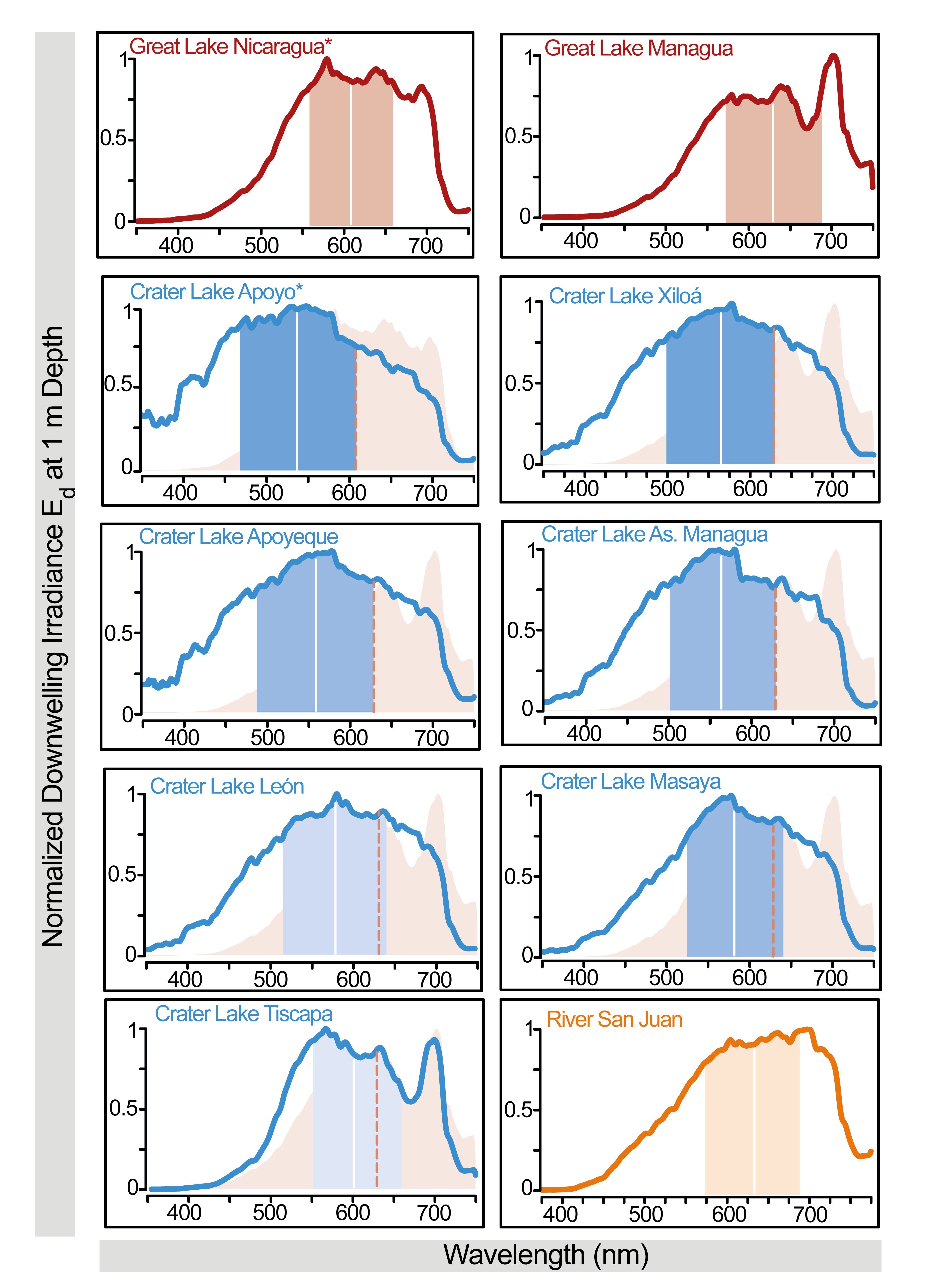


**Figure S1**. Reference spectral curves showing the normalized downwelling irradiance (E_d_) at one meter depth for each site. Vertical solid lines below each spectral curve represent the spectrum-halving wavelength λP50 within the colored area depicting the spectral bandwidth, where 50% of the photons are found. The intensity of the spectral bandwidths represents the relative luminosity at one meter depth (%E_d_), with darker colors meaning higher %E_d_. The shaded area displayed in the background of the crater lake inserts shows the photic environment plus λP50 (dashed line) of the respective source great lake based on Midas cichlids’ colonization history (Kautt et al., 2016; 2018; 2020).


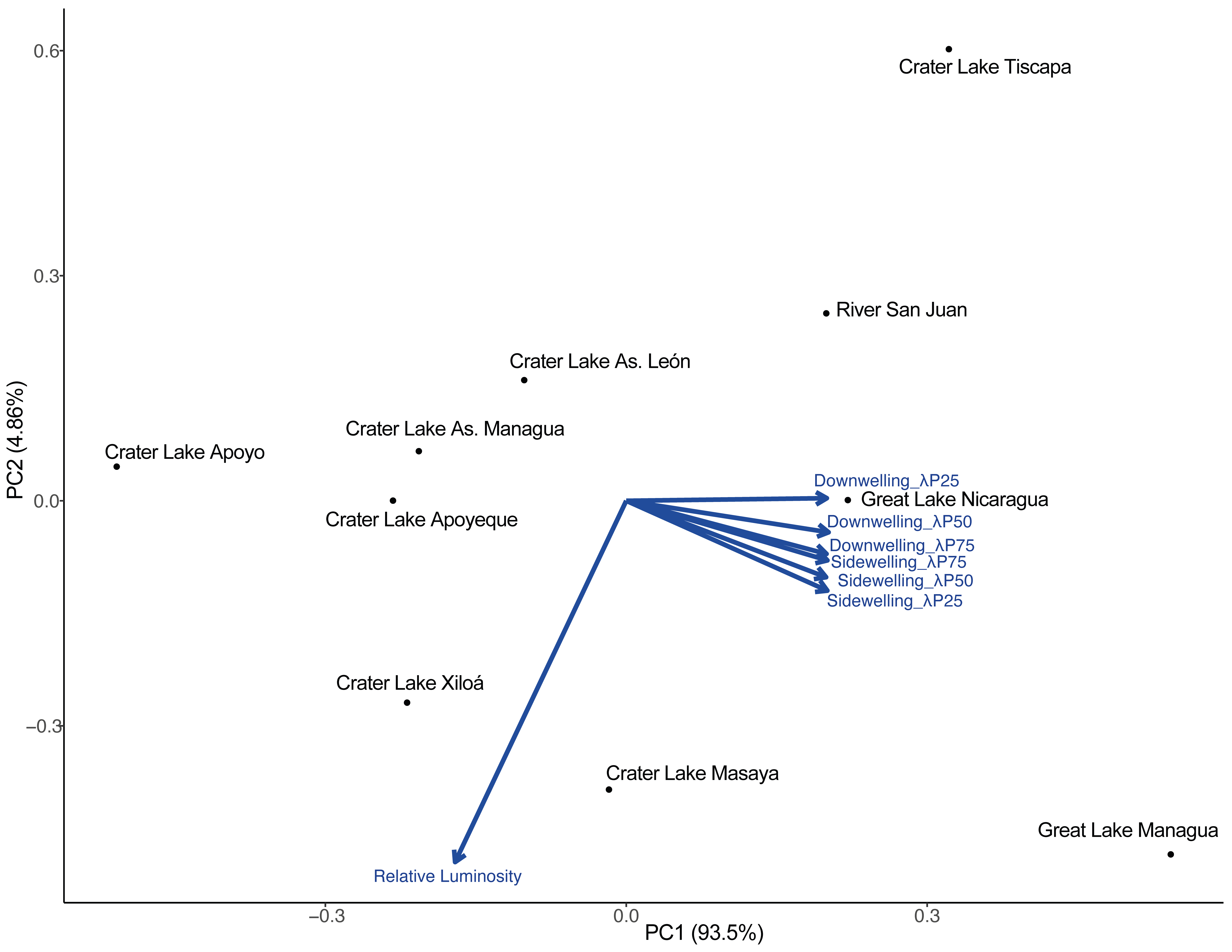


**Figure S2.** Biplot depicting the PCA scores generated from photic variables from all sites at one meter depth where fish were collected (Table S1). Arrows represent the loadings of each variable.


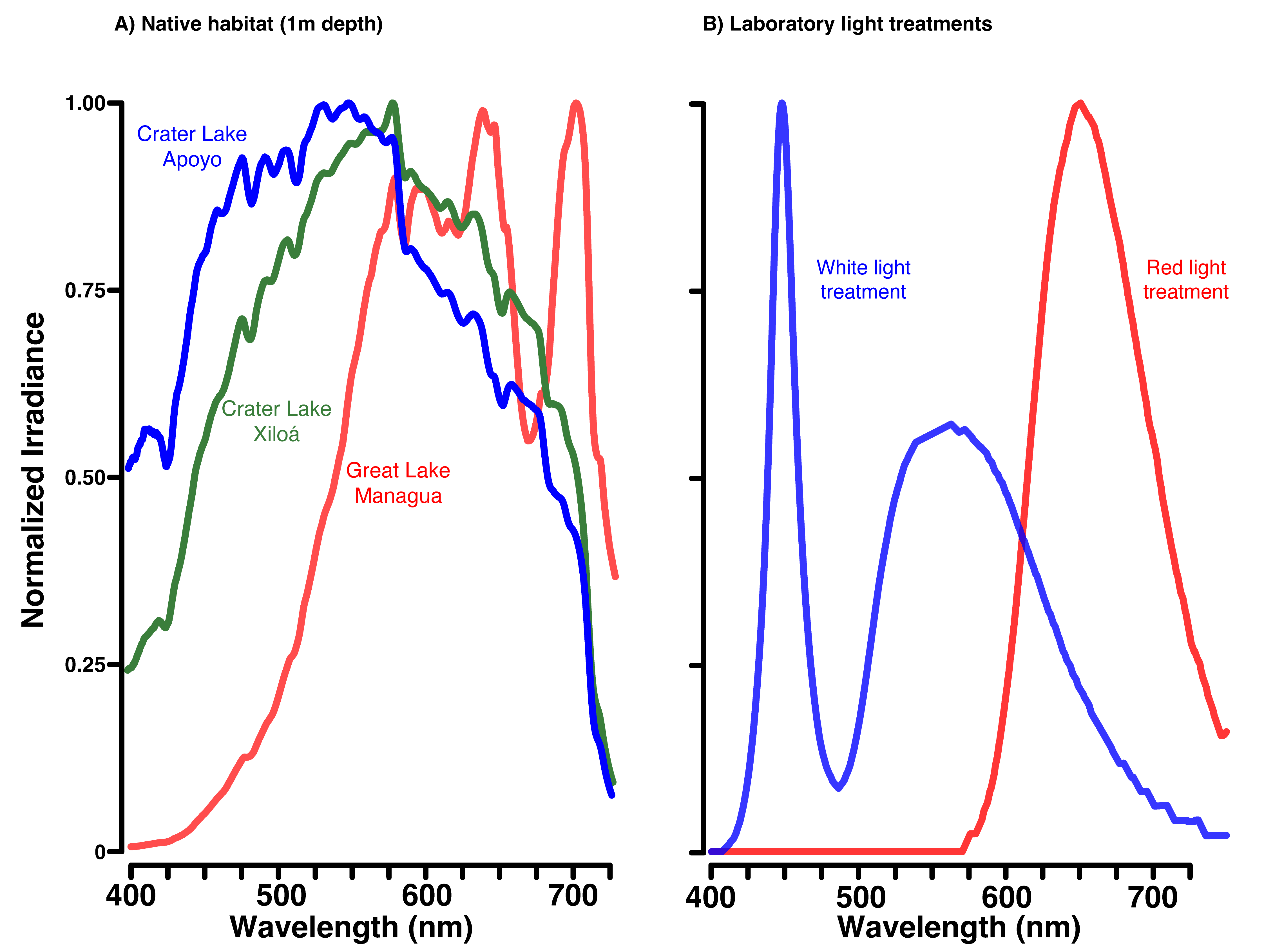


**Figure S3.** (A) Normalized downwelling irradiance at 1m depth in crater lakes Apoyo (blue) and Xiloá (green) and in great lake Managua (red) from Bertinetti et al., 2023 (B) Normalized downwelling irradiance used in the laboratory for white light treatment (blue) and red light treatment (red).


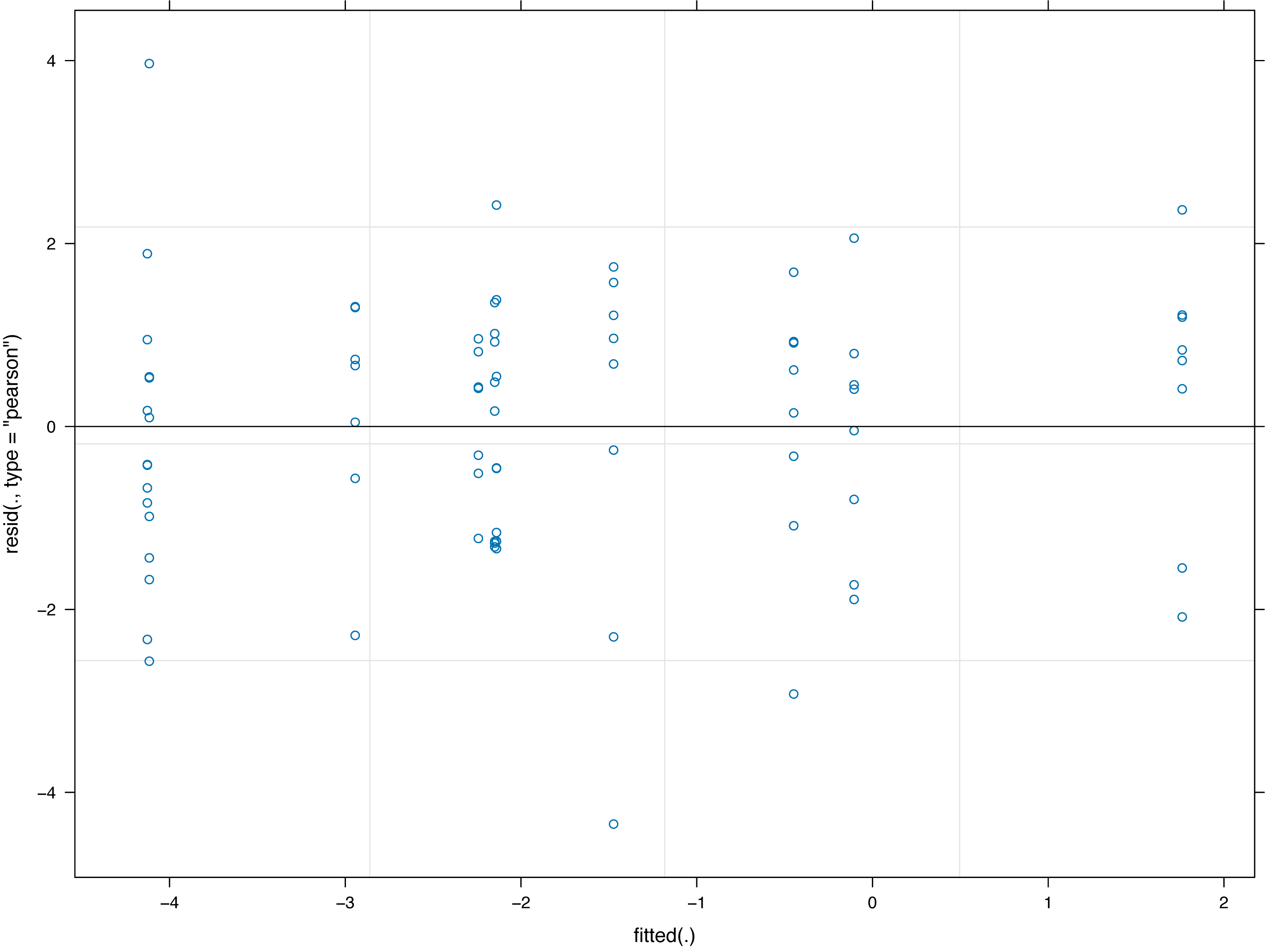


**Figure S4.** Diagnostics plot for linear mixed-effects model using PC1 as predictor variable of log-normalized relative *cyp27c1* expression with location as a random effect (Fig. 1)

**References:**

Bertinetti, C., A. Härer, N. Karagic, A. Meyer & J. Torres-Dowdall, 2024. Repeated Divergence in Opsin Gene Expression Mirrors Photic Habitat Changes in Rapidly Evolving Crater Lake Cichlid Fishes. The American Naturalist 203: 604-617 https://doi.org/10.1086/729420.

Kautt, A. F., C. F. Kratochwil, A. Nater, G. Machado-Schiaffino, M. Olave, F. Henning, J. Torres-Dowdall, A. Harer, C. D. Hulsey, P. Franchini, M. Pippel, E. W. Myers & A. Meyer, 2020. Contrasting signatures of genomic divergence during sympatric speciation. Nature 588: 106-111 https://doi.org/10.1038/s41586-020-2845-0.

Kautt, A. F., G. Machado-Schiaffino & A. Meyer, 2016. Multispecies Outcomes of Sympatric Speciation after Admixture with the Source Population in Two Radiations of Nicaraguan Crater Lake Cichlids. Plos Genetics 12: e1006157 https://doi.org/10.1371/journal.pgen.1006157.

Kautt, A. F., G. Machado-Schiaffino & A. Meyer, 2018. Lessons from a natural experiment: Allopatric morphological divergence and sympatric diversification in the Midas cichlid species complex are largely influenced by ecology in a deterministic way. Evolution Letters 2: 323-340 https://doi.org/10.1002/evl3.64.
